# Disparity of time-contrast curves generated by various types of power injectors used in magnetic resonance imaging

**DOI:** 10.1038/s41598-020-76536-x

**Published:** 2020-11-11

**Authors:** Marcus Doppler, Ewald Moser, Uros Klickovic, Christian Nasel

**Affiliations:** 1 Department of Radiology, University Hospital Tulln, Alter Ziegelweg 10, A – 3430 Tulln a.D. Donau, Tulln, Austria; 2 Center for Medical Physics and Biomedical Engineering, Medical University of Vienna, Vienna, Austria

**Keywords:** Magnetic resonance imaging, Medical imaging

## Abstract

The profiles of time-contrast (TC) -curves from popular MRI injectors derived at the injection site of the attached tube-line system were compared. Variations of TC-profiles were previously reported to potentially influence image quality in time critical MRI measurements. TC-curves from five injectors obtained during commonly used injection protocols were assessed according to representative quality criteria: (1) correlation strength between a fitted boxcar function and the TC-curve (cBCF) and (2) difference between true and expected injection time (dBIT). Additionally, the impact from technical injector properties: pump type, line volume, maximum injection power and type of contrast medium (CM) on the TC-profiles was evaluated. Injectors using a piston-syrinx (PS) mechanism for CM-injection performed significantly better than those working with a peristaltic roller pump (RP) technique. Besides injection mechanism, line filling volume showed a strong influence on the final TC-curves, where larger filling volumes induced worse cBCF- and dBIT-results. Therefore, to achieve an optimal bolus in clinical MRI use of a PS-injector seems recommendable. Besides their pump mechanism, RP-injectors appeared additionally hampered by their high volume line systems, pointing out an unfavourable coinicidence of these technical features in RP-injectors. This should be considered, particularly, in comparative or time-critical MRI-studies.

## Introduction

Reproducible shape and gradient of an administered contrast medium (CM) bolus is mandatory for various magnetic resonance imaging (MRI) protocols. For instance, in functional, dynamic MRI (DC-MRI) or MR-angiography (MRA) examinations use of power-injectors was recommended to warrant constant bolus properties^1,2^. However, when using a CM-injector, implicitly an optimal and comparable contrast bolus definition for all injector models, equipped with their different tubes and lines, is assumed. Though intra-corporal factors of a patient also significantly modify the CM-bolus, technical properties of the various injectors, like injection technique and pressure, are also known to play an important role^3–5^. Thus, it is mandatory to understand the course of the effective time/contrast (TC) curve of the CM-bolus finally administered to a patient, since this is the basis for all further transformations of the CM-bolus. Especially, the injection mechanism, where injectors are either of piston-syringe (PS) or roll-pump (RP) type, and the dimensions of the mounted lines and tubes vary considerably between the different power injectors. Therefore, we hypothesised that the TC-curves of the finally injected boluses may also vary substantially, thereby rejecting the general assumption of an optimal and comparable CM-bolus definition by all injector models.

## Results

### Correlation of photometric TC-curves with fitted boxcar function (cBCF)

**Figure 1:**
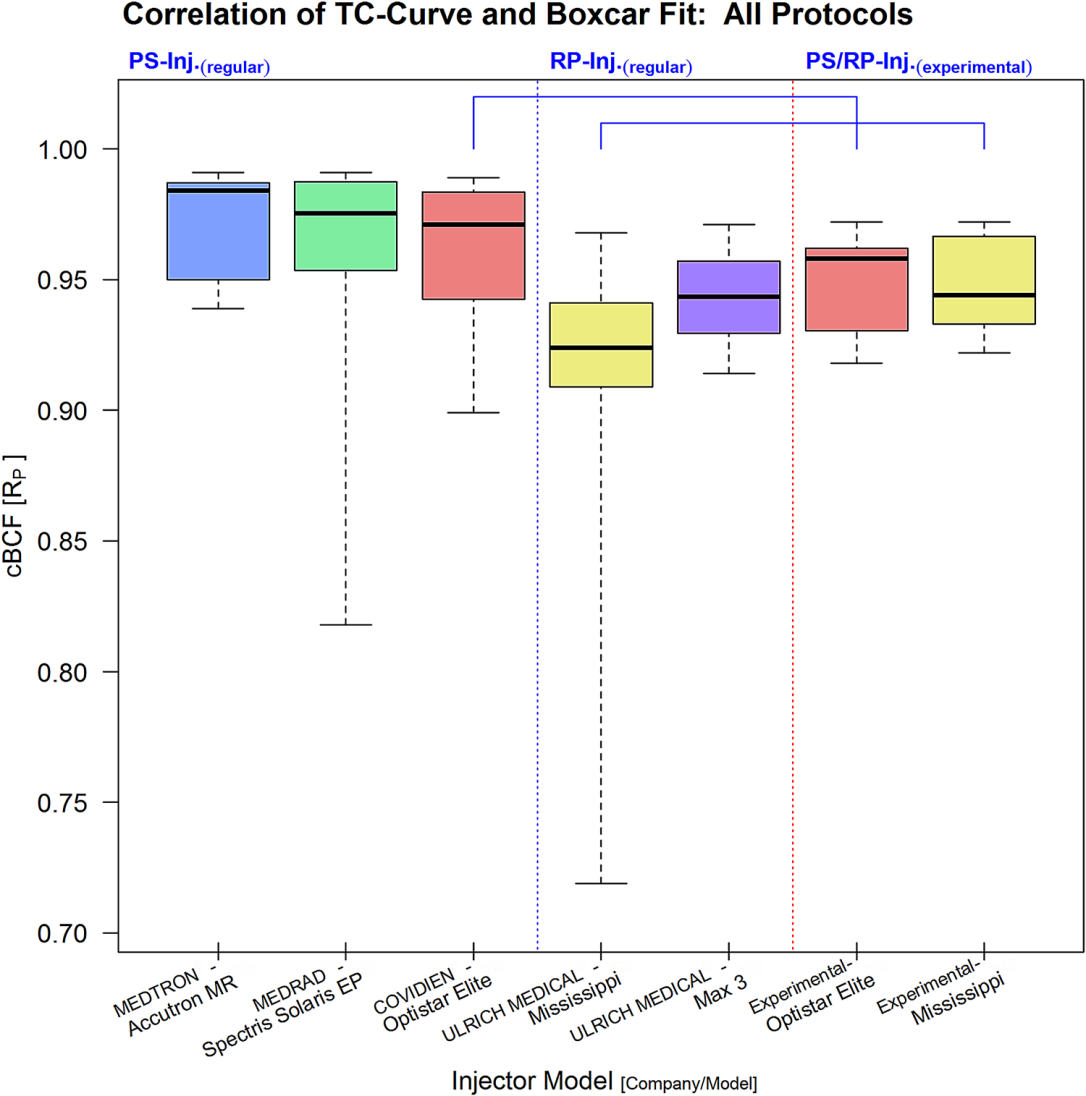
Cumulatively testing all injection protocols showed a significantly stronger cBCF ($R_{P}$, Pearson) for PS-injectors (left section, separated by vertical blue dotted line) equipped with regular lines. The cCBF for regularly equipped RP-injectors was significantly lower (middle section, between vertical blue and red dotted lines). An experimental exchange of high and low volume tube line systems between a PS- and a RP-model (right section, separated by vertical red dotted line) showed some improvement of cBCF in the RP-model, while cBCF in the PS-model declined (both not significant; horizontal brackets indicate injector ties).

PS-type injectors showed significantly stronger cBCFs than RP-injectors (DTK-test: $R_{P}$-cBCF; p < 0.05) in cumulative assessment of all injection protocols (Fig. 1). In the sub-group analysis (supplementary figure S1) PS-injectors performed significantly better in the vVol- and cVol-group, with only moderate differences in the cVol-protocol group, where the CovOpt PS-injector failed to reach a significant result (DTK-test: $R_{P}$-cBCF; [vVol]: p < 0.001, [cVol]: p < 0.01). In the mVol-group, only the MedAcc PS-injector achieved a significantly stronger cBCF than the RP-injectors (DTK-test: $R_{P}$-cBCF; [mVol]: p < 0.05). Experimentally exchanging high and low filling volume tube lines between PS- and RP-injectors did not significantly alter their performances, but led to a measurable deterioration of cBCF of the PS-injector, while with the RP-type system only small improvements were observed (DTK-test: $R_{P}$-cBCF; [all-exp]: n.s.). A full summary of all cBCF results is given in Table 1.

**Table 1:**
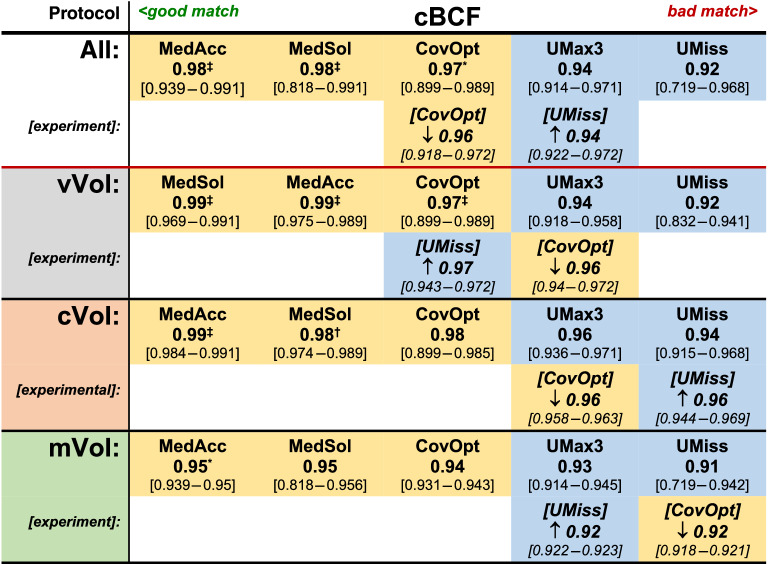
Correlation matrix.

Generally, PS-injectors (yellow fields) showed stronger correlations between the fitted boxcar function and the measured TC-curve profiles (cBCF; Pearson correlation coefficient: $R_{P}$) than RP- injectors (blue fields). The cBCF-results (median and [range]) of the various protocol groups (all, vVol, cVol, mVol) are shown for all injector models. In experimental testing ([experiment]) arrows indicate improvement (↑) or deterioration (↓) compared to the result from the correlated regularly equipped injector model. All findings are arranged in rank order from left (best result) to right (worst result). Significant results were tagged (DTK-test [corr.]; sig. levels: *p < 0.05, ^†^p < 0.01, ^‡^p < 0.001).

### Difference between effective and expected bolus injection time (dBIT)

**Figure 2:**
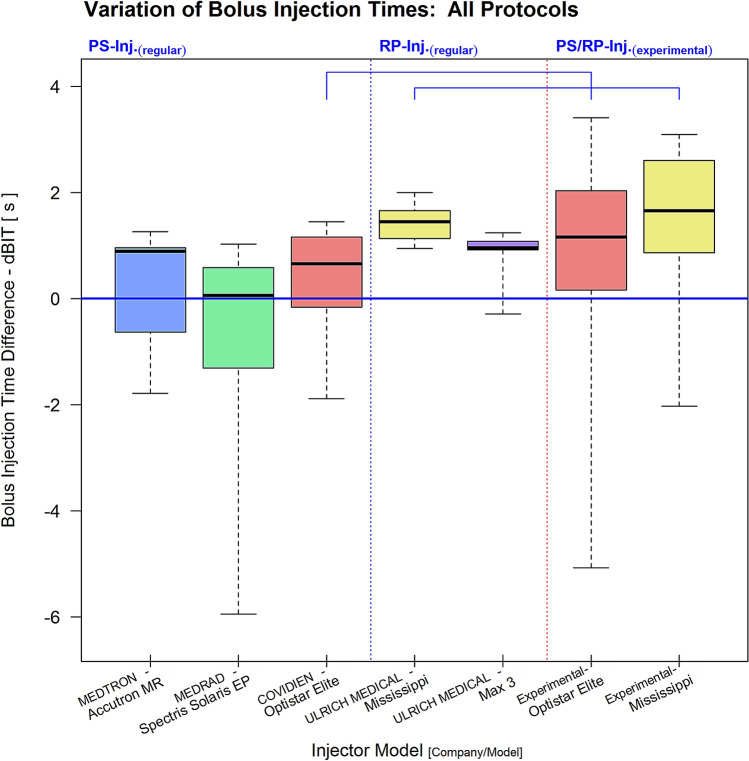
Cumulative assessment of all injection protocols showed clearly lower variations of the true injection time with regularly fitted PS-injectors (left section, separated by vertical blue dotted line) compared to RP-injectors (middle section, between vertical blue and red dotted lines). Experimental attachment of a high volume line to a PS- and a low volume one to a RP-injector (right section, separated by vertical red dotted line) led to deterioration of the performance of both injector types (both not significant; horizontal brackets indicate injector ties).

Concerning dBIT, in the cumulative assessment of all injection protocols (Fig. 2) PS-injectors also achieved better results than the RP-injectors (DTK-test: dBIT; [all]: p < 0.001). In addition, comparing the two RP-injector systems, dBit of the UMax3 model was significantly lower than that of the UMiss injector (DTK-test: dBIT; [all]: p < 0.05). In the sub-group analysis (supplementary figure S2), dBit of the MedSol PS-injector was significantly lower than that of all other injectors in the vVol-group (DTK-test: dBIT; [vVol]: p < 0.001). Notably, the UMax3-injector reached again a significantly better dBIT compared to its RP-injector counterpart, the UMiss-system (DTK-test: dBIT; [vVol]: p < 0.05). In the cVol-group the CovOpt (p < 0.05) and the MedSol (p < 0.001) PS-injectors performed significantly better than all other models (DTK-test: dBIT; [cVol]). In the mVol-group dBIT of the MedAcc (p < 0.01) and MedSol (p < 0.001) PS-injectors were significantly lower compared to the RP-injectors (DTK-test: dBIT; [mVol]). For the rest, dBIT was comparable within this group. Except for the mVol-group, the experimental exchange of tube line systems between PS- and RP-injectors generally deteriorated dBIT of the PS- and slightly improved that of the RP-injector. In the mVol-group this exchange led to some improvement with both injector systems. However, none of these changes was significant (DTK-test: dBIT; [all-exp]: n.s.). A full summary of all dBIT results are listed in Table 2.

**Table 2:**
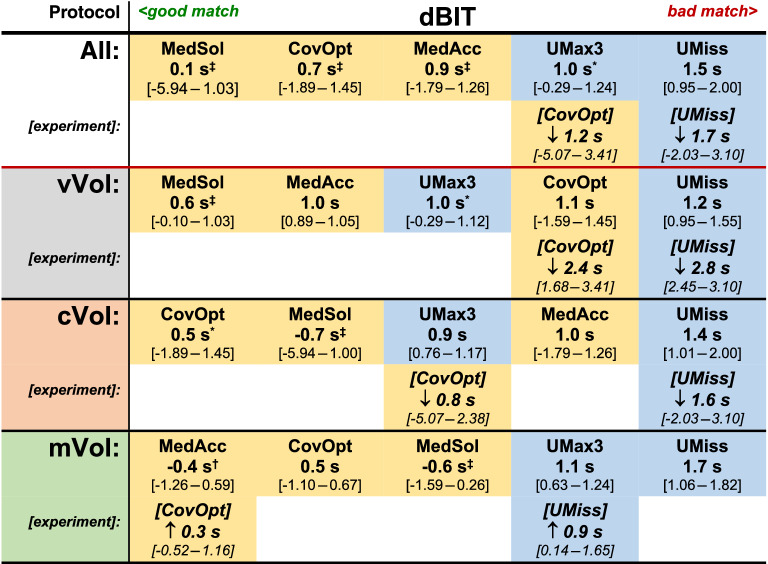
Time differences between true and expected injection time (dBIT; unit: seconds) were generally smaller with PS- (yellow fields) than with RP- (blue fields) injectors in the various protocol groups (all; vVol; cVol; mVol).

Results for dBIT (median and [range]) are displayed for each injector model, where in experimental testing ([experiment]) arrows indicate improvement (↑) or deterioration (↓) compared to the correlated regular result. All findings are arranged in order of ranks from left (best result) to right (worst result). Significant results were tagged (DTK-test [corr.]; sig. levels: *p < 0.05, ^†^p < 0.01, ^‡^p < 0.001).

### Covariates of cBCF and dBIT

Analysis of potential covariates of cBCF revealed the factors pump type (p < 0.0002) and filling volume of the tube line (p < 0.009) as direct confounders of cBCF. Type of CM and injection power (defined by the maximum injection pressure possible) showed no significant influence on cBCF, though, significant interactions between injection power and pump type (p < 0.0002), as well as, tube line volume and pump type (p < 0.023) were found (AnCova; cBCF: flow rate = 5.0 ml/s & injection volume = [2.0–15.0 ml], n = 94).

For dBIT the factors pump type (p < 0.004), injection power (p < 0.005), and CM-type (p < 0.03), were identified as direct confounders. Additionally, significant interactions between tube line volume and pump type (p < 0.0001), CM- and pump type (p < 0.02) and CM-type and injection power (p < 0.04) were found (AnCova; dBIT: flow rate = 5.0 ml/s & injection volume = [2.0–15.0 ml], n = 94). Generally, we noticed a significantly higher chance to achieve better compliance with our quality criteria with all protocols when Gadoterate was used for the injections (odds ratio—test; p = 0.035).

## Discussion

Comparing five commercial MRI-injector systems we obtained evidence that, depending on the respective injector type and the mounted tube line system, CM-boluses differed significantly in their shape and effective injection time at the site of injection at the patient. As previous reports suggested a tight relation between the CM-bolus characteristics at the injection site and image quality^1,2,5^, it is conceivable that measures obtained even with the same imaging- and injection-protocol could lack consistency when different injector models are used.

**Figure 3:**
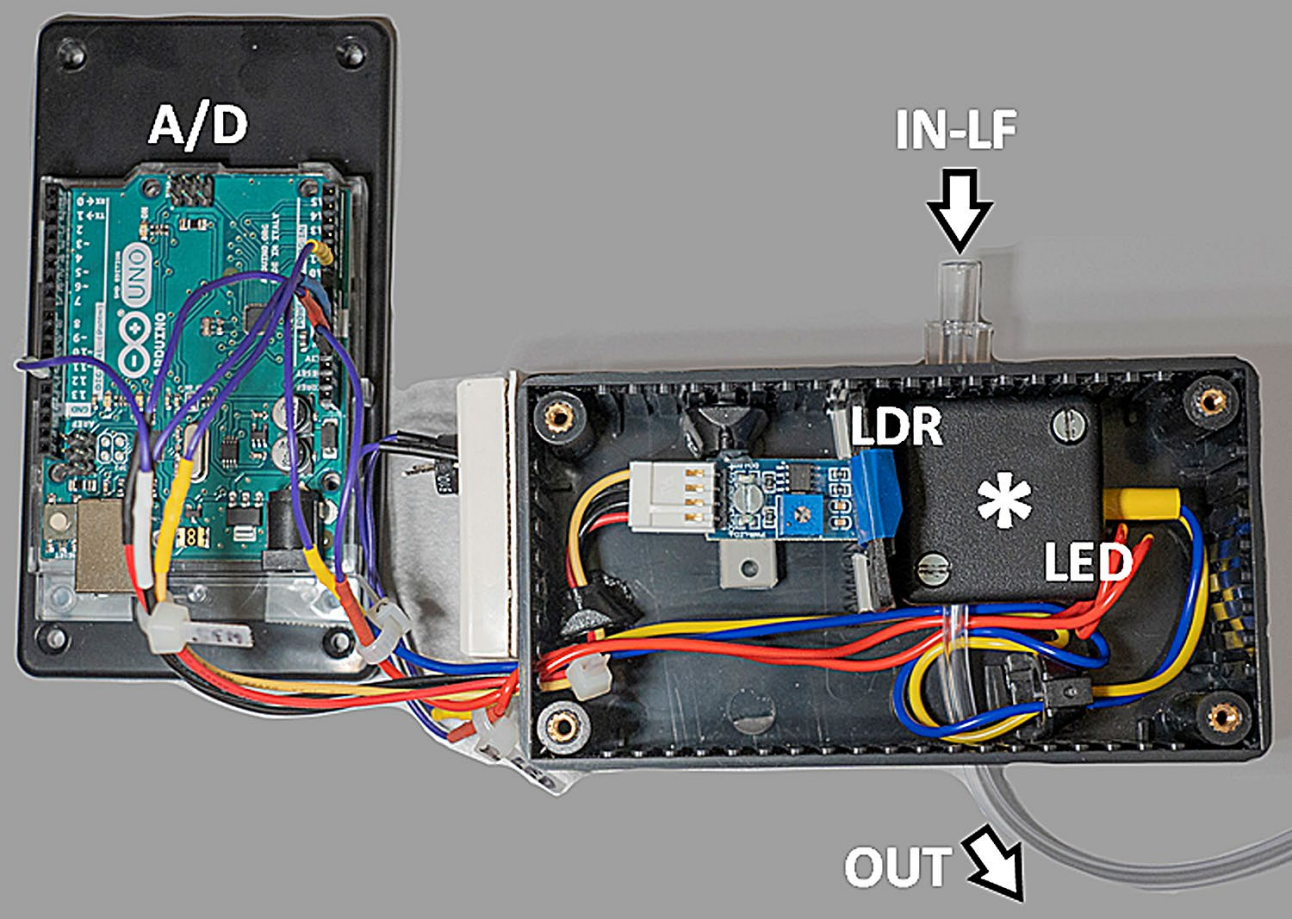
The sensor box used for the photometric assessment was a double case box with the actual photometric chamber (white asterisk) placed inside the outer box to exclude influences from ambient light. Injectors were connected via the Luer-lock fitting at the in-flow socket (IN-LF). Inside the photometric chamber a light dependent resistor (LDR) and a power light emitting diode (LED) were placed vis-à-vis around the transparent probe line with apertures fitted to the cell to additionally exclude interference with scatter light. Fluid that passed through the sensor box was drained (OUT) immediately to a reservoir (not shown). The analog/digital converter (A/D) transmitting all data to the PC workstation was placed outside, on top of the outer box.

Similar to variations caused in manual injections^2^, the various injector models exhibited meaningful disparities of their bolus TC-curve profiles in our study, depending on their technical features. This includes maintenance of steady-state flow or injection pressure, or even different dimensions of the attached tube line system. Analogous to the clinical injection site, TC-curves were obtained photometrically at the tube line end of five commercial injectors in this study and examined for their compliance with quality standards predefined as cBCF and dBIT (Fig. 3). As we primarily aimed to demonstrate relevant differences of the generic bolus formation by the respective injector, all injectors were tested under laboratory conditions without connection to a patient and devoid of any pressure load, e.g., from venous cannulas etc.. This approach was chosen to avoid additional uncalculable bias from geometric deformation of the attached tube line systems during the injections, since all injectors came with lines of different lengths and wall thicknesses.

The cBCF criterion describes the strength of correlation between a photometric TC-curve and the boxcar function fitted to this particular curve using the Pearson correlation coefficient $R_{P}$. Thereby, the TC-curve of an optimal bolus was considered to conform to a boxcar function, because steeply sloped flanks representing short wash-in and -out phases with a flat peak plateau phase in between, corresponding to constant CM-flow during the actual injection, were expected^6^. Additionally, the effective injection time was estimated from the duration of the peak plateau phase derived from the fitted boxcar model. Hence, the dBIT criterion was calculated as the difference between effective and protocol specific expected injection time. In case of a good match between the injection protocol parameters, the TC-curve, and the boxcar model, dBIT should be minimal and cBCF maximal (Figs. 1, 2).

**Figure 4:**
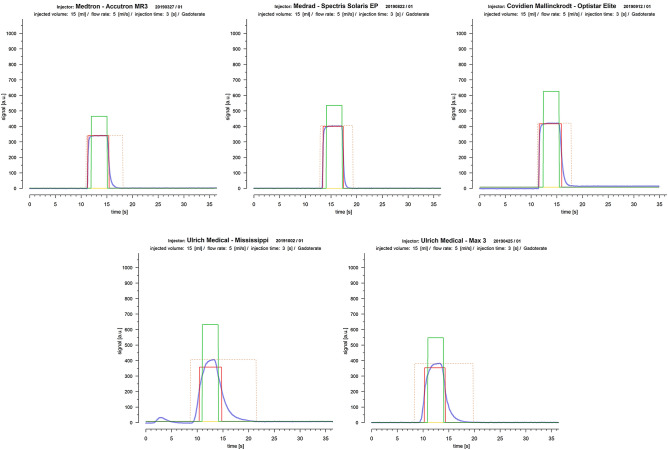
Examples of TC-curves (solid blue lines) derived from PS- (first row) and RP-injectors (second row) all obtained during injection of 15 ml of Gadoterate at a flow rate of 5 ml/s with regular equipment. The shapes of TC-curves gained by PS-injectors were clearly closer to the boxcar profile (solid red lines: fitted boxcar function) than those from RP-injectors with also stronger cBCF-correlations found for PS-injectors. All injectors failed to exactly reach the programmed injection time of 3 s (solid green lines: box car function strictly applying to injection parameters), though dBIT was in favour of PS-injectors. The dashed yellow lines mark the range necessary to cover the full TC-curve of each bolus, where this range was also clearly wider with RP- than with PS-injectors.

**Figure 5:**
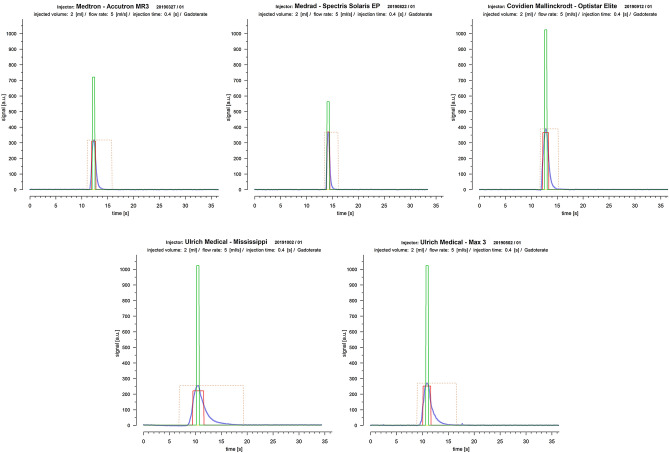
Injecting very small CM-volumes reduced the measured TC-curves (solid blue lines) to a short wash-in and –out phase only by condensing the interposed plateau phase of the expected boxcar function (solid red lines: fitted boxcar function) to a single peak. Though, generally cBCF declined, PS-injectors (first row) still performed better than RP-injectors (second row). With PS-injectors true and expected (0.4 s) injection times nearly matched, while with RP-injectors the expected injection time was clearly exceeded (solid green lines: box car function strictly applying to set injection parameters). Dashed yellow lines mark the range necessary to cover the full TC-curve of each bolus, which was clearly wider in RP- than in PS-injectors.

Generally, the match between measured TC-curves and the fitted boxcar function was found acceptable, thus, confirming the practicability of our quality criteria (Figs. 4, 5). However, significant differences between the various injector systems with a tight relation to their differing technical properties were found for both criteria, cBCF and dBIT, respectively. In order to discern possible sources explaining the observed differences of cBCF and dBIT more closely, several injection protocols simulating applications of routinely used very small and also, realistic, large volumes with varying high and low flow rates were investigated. Analysing cBCF across all protocols with respect to the various technical injector features revealed that TC-curves from PS-injectors conformed significantly better to the postulated ideal boxcar shape than RP-injectors. This difference remained significant, independently from whether the CM-injection volume (cVol-protocol group) or the flow rate (vVol- protocol group) was kept constant during the injections (supplementary figure S1). Nevertheless, the effect size towards better a performance of PS-injectors was bigger when a constant flow rate of 5 ml/s was used, but almost vanished in protocols (mVol-protocol-group) with injection of very small CM-volumes (2.0 ml). Since dBIT depends on cBCF, across all protocols the behavior of dBIT was similar to, but in its effect weaker than, that of cBCF (Figs. 1, 2). Accordingly, PS-injectors showed a significantly better compliance with the programmed injection time than RP-injectors, which was also true for the various protocol sub-groups (supplementary figure S2). However, deviations from the expected injection times were bigger with injections of very small CM-volumes (~ 2.0 ml). Combined with the cBCF-results, this suggests a limited advantage using a power injector for the administration of vary small CM-volumes, like in examinations of young children. Anyway, even with injection of small CM-volumes PS-injectors reached a better compliance with our quality criteria than the RP-systems (Fig. 5). This agrees very well to other studies investigating the impact from different pump mechanisms on the final bolus definition in clinical CT, where significantly greater variances of steady-state flow potentially altering the bolus shape were demonstrated for RP-type injectors compared to PS-type models^5^. Therefore, the injection technique used to propel CM to the injection site at the patient seems to possess the strongest influence on the TC-curve profile, which was also confirmed by the analysis of covariates in our study.

Besides pump type, additionally the filling volume of the attached tube line system, the injection power and the CM-type significantly influenced the final TC-curve profile. While cBCF was more affected by the line filling volume, dBIT was additionally influenced by injection power and CM-type, where also tight interactions between the various confounders were found. This seems related to the fact that not only the RP-injection technique appeared suboptimal for injection conditions as given in clinical MRI, but also the RP-injectors had to move an unfavourably higher line filling volume. Bigger filling volumes potentially increase dilution of the CM-bolus pushed forward by saline used as propellant, thereby leading to a flattening of the expected steeply sloped flanks of the TC-curve profile representing the wash-in and -out phases. This assumption was supported by significantly weaker cBCFs and increased dBITs found with RP-injectors in all investigated protocols. As the tube line volume was found to influence the TC-curve profiles independently of the injection technique, with all pump types cBCF was weakest with injections of very small CM-volumes where, obviously, the whole TC-curve profile of the short bolus formation became quickly altered by dilution (Fig. 5). Nevertheless, even with these protocols PS-injectors performed best.

The behaviour of dBIT was similar to cBCF, but in addition to pump type and line volume, to a lesser degree, also injection power and CM-type significantly influenced the measurements. This was probably owed to the fact that the two CM compounds used in this study had distinctly different and rather high viscosities (Gadoterate: 3.4 mPA·s [@20°]; Gadoteriol: 2.0 mPA·s [@20°]; water: 1.0 mPA·s [@20°]^7^), as compared to water. This, generally, favours injectors with higher injection power. Throughout all protocols the chance to get the best compliance with cBCF and, consequently, dBIT was significantly higher when Gadoterate was used, although in absolute numbers the encountered differences were too small to achieve statistical significance. Anyhow, this suggests that, besides pump type, dilution between the saline propellant and CM could play a more relevant role in high volume tube line systems compared to low volume ones. The higher viscous Gadoterate-bolus is expected to be more robust to dissolution by the saline propellant acting as a solvent, than the low viscous Gadoteridol-bolus, since both processes engaged, dissolution and diffusion, depend on the diffusion coefficient, which in turn is related to viscosity according to the Stokes–Einstein equation^8^. As the contribution of this dissolution effect to the final TC-curve profile was not significant, we abstained from modelling the intermixture of solvent and solute in the tube line systems and, also, refrained from further simulations.

Nevertheless, the pump type remained the strongest effect on the TC-curve profile, which was proven by our experiment, where high and low volume tube lines were exchanged between a PS- and a RP-injector model. Note that no patient was connected in all our measurements and that in clinical MRI this must not be done anyway. While cBCF of the PS-model decreased, with relatively small effect on dBIT, both quality criteria showed only some improvement with the RP-model. Moreover, the performance of the regularly equipped PS-model was never reached by none of these injector configurations. Therefore, it seems conceivable that optimising the tube line volume of RP-injector systems could indeed improve their performance, but for the moment our findings imply that this cannot overcome their apparently suboptimal injection technique.

However, several limitations have to be considered. First, fitting a boxcar function to the TC-curves was decided empirically. Though other model functions might have also yielded a good result, a relevant change of findings from PS- and RP-injectors is unlikely. The boxcar function offers all components required from an ideal CM-bolus with steep flanks and a constant plateau phase simulating the major injection phase. Since we achieved rather high correlations with the various TC-curves, even if the boxcar function was not the absolutely perfect model function, it excellently served the task. Second, all TC-curves were measured without pressure load from a venous cannula normally used in clinical MRI. An additional pressure load on each injector would have probably modified the shape of the TC-curves. Since the mixture of CM and saline should widely resemble a Newtonian fluid, a resistor added at the end of the line, comparable to a venous cannula, would have induced the most significant change into the measurements. In principle, an additional elastic deformation of the various line systems could also affect cBCF and dBIT, but a stronger effect than from the active cross section of the end-line restor is unlikely. As, therefore, all injectors would have been affected in the same way, a significant change or even an improvement in the relations between PS- and RP-injectors is not to be expected. Nevertheless, in case of the CovOpt-model, which offered the lowest injection power of all PS-injectors, this assertion remains speculative and further studies on this topic are required. Third, we just tested a small selection of injectors available to us, which does not allow generalisation. However, all tested injectors are very well established systems in clinical routine MRI. Thus our data should offer an acceptably representative overview for radiologists and technicians engaged with contrast enhanced MRI examinations.

In conclusion, all injectors systems assessed in this study reached acceptable results when testing injection protocols common in clinical MRI, whereas the injector performance was generally decreased with injections of very small CM-volumes. However, independently of the investigated protocol type PS-injectors performed significantly better than comparable RP-injectors working with a peristaltic roll pump mechanism. Analysing the various technical injector properties suggested an unfavourable coincidence of an apparently suboptimal injection mechanism with tube line systems dimensioned too big in RP-injectors. As this could potentially affect results, particularly, from dynamic and time critical CM-enhanced MRI-examinations, especially in comparative studies, an influence of the respective injector type should be considered. Further trials to investigate the full impact of the various technical parameters of clinically employed power injectors on clinical MRI studies are required.

## Materials and methods

### Tested injectors

In this study, we explored five commonly used MRI-injector models. The injector pumps included three different PS- and two RP-injector systems:

The PS-injector MEDTRON Accutron MR (MedAcc; MEDTRON AG, Saarbruecken, Germany) was equipped with two syringes, one for CM and one for saline, each with a capacity of 200 ml. Injector valves were located immediately at the syringes and at the junction of the syrinx-lines. Another valve was located at the end of the patient line.

The PS-type MEDRAD Spectris Solaris EP(MedSol; MEDRAD, BAYER MEDICAL CARE BV, Maastricht, The Netherlands) injector is equipped with two syringes of different volume. The larger one is used for saline with a volume of 115 ml. The smaller syringe contained the CM and has a capacity of 65 ml. Two valves were placed at the exit of each syringe, with no further valve in the patient line.

The third PS-type injector tested was the COVIDIEN Optistar Elite (CovOpt, built: 2014; formerly: COVIDIEN Deutschland GmbH, Neustadt/Donau, Germany; now: GUERBET, Roissy CdG Cedex, France) system which has also two syringes mounted with a capacity of 60 ml each. On the CovOpt injector two valves were placed at the syringes and a third one in the patient line about 50 mm after the junction of the syrinx-lines.

The first RP-injector, the ULRICH Mississippi (UMiss; ULRICH GmbH & Co.KG, Ulm, Germany), sucks CM and saline directly from the flasks mounted at the machine, where no valves were interposed between the containers and the roll pump box. The patient line exiting the pump box includes one valve at its very end.

The ULRICH Max 3 (UMax3; ULRICH GmbH & Co.KG, Ulm, Germany) was the second, and, as compared to the UMiss, the more recently designed RP-injector tested in this study. This model also receives CM and saline directly from a flasks mounted at the system without any valve at this part of the line. Again, similar to the UMiss-model, there is a valve at the end of the patient line.

Note that all injector systems came with regular tube-line systems approved for the task by the respective vendor. Thus, different specifications concerning their inner diameter, length and, in consequence, filling volume are evident. The actual specifications are summarised in Table 3. All injectors and tube-line systems were made available freely by the respective vendor upon request, except for the UMiss and the CovOpt injectors, which had already been used at our department for several years.

**Table 3:**
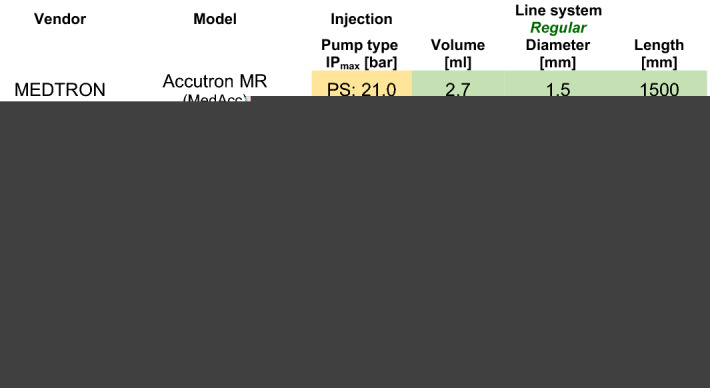
List of technical details of PS- (yellow fields) and RP- (blue fields) injector models tested in this study showing the highest injection pressure (IP_max_), as well as the dimensions of the attached tube line system.

Regular system configurations (green fields) conformed to vendor approved standards. Not approved experimental configurations (red fields) served only to explore an influence from the line filling volume on the measurements.

### Photometric equipment and measurement

Shape, gradient and true bolus injection time of all TC-curves were measured photometrically using a home-built photometric cell equipped with a high power light emitting diode (LED, THOMSEN LED-5–40.000 W, white, round 5 mm, 40,000 mcd 8°, 30 mA, 3.1 V; CONRAD Electronic GmbH & Co KG, Wels, Austria) and a photosensitive light dependent resistor (LDR) control sensor module (LM393), which were connected to an analog–digital converter board (A/D-C, ARDUINO Uno). The series resistor for the LED in the light emitter circuit was chosen adjustable to enable correct triggering of the baseline. LED and LDR were arranged vis-à-vis with the probe line for the stained CM passing in between. Shutter systems were built in to avoid bias from scattered light and, additionally, the whole photometric cell was placed inside a double case box to prevent any influences from ambient light. The probe line was equipped with a Luer-Lock fitting (female) immediately at the sensor box, where the respective injector lines were connected to. The effective diameter of the tube in the photometric chamber was set to the largest line diameter of the injector sample to avoid any influence on the bolus shape from an additional flow resistance. The probe line length between photo sensor and fitting was about 15 mm (Fig. 3). The photometric data from the sensor box were sampled at a frequency of 12.2 Hz and recorded via the A/D-C. All data were transferred in real time to a PC-workstation (HP Z440 – INTEL Xeon E5-1680 v4 processor (8 cores), HEWLETT PACKARD Austria GmbH, Wien, Austria), where the data was stored in csv-file format for further assessment. During an experiment photometric data were simultaneously displayed graphically at the PC work station to allow real time control of the respective injection and to verify a stable baseline. Photometry and data handling were performed using in-house assembled and developed hard- and software (C.N.). The A/D-C sketch used was compiled using the ARDUINO IDE (version 1.8.12), while storage and visualisation of the recorded data were implemented as web-based server-client solution on the PC-work station employing scripts written in HTML5 and nodeJS JavaScript language (version 4.2.3) incorporating the ‘D3.js’-module. For software development the Netbeans IDE (version 8.0.2) was used^9–12^.

Gadoterate (Dotarem, GUERBET, France) and Gadoteridol (ProHance, BRACCO, Italy), respectively, were alternatively used as CM for the various injection protocols. Vials containing 100 ml CM were stained with 2 ml of a Methylthioninium Chloride solution with a concentration of 10 mg colorant/ml (Metiblo, LABORATORIES STEROP, Brussels, Belgium). The dyed CM vials were swivelled carefully until a homogeneous solution of the colorant was reached. Stained CM and an appropriate quantity of unstained saline were filled into the respective injector reservoir. Then the injector was attached to the sensor box using only originally supplied syringe- and patient-lines approved as '*standard for task*' by the vendors. Each time, before the start of recordings of a particular CM-injection, all lines including the sensor box were flushed with saline until a stable baseline was reached. Introducing a 10 s CM injection delay enabled acquisition of sufficient baseline data points for further assessment. Recording was ceased when the photo-densitometric TC-curve had returned to baseline again, thereby allowing a variance of maximum two standard deviations of the original baseline.

### Injection protocols, protocol-groups and experiments

Different injection protocol groups were employed for all injector models. In the first protocol group the injected CM volume was varied at a constant flow rate of 5 ml/s. In this way, volumes of 15.0, 10.0 and 5.0 ml of both CMs, i.e., Gadoterate and Gadoteridol, respectively, were applied via the sensor box with expected bolus injection times at the sensor of 3.0, 2.0 and 1.0 s (variable volume group: vVol). The second injection protocol group observed the bolus shapes of constant injection volumes (10 ml) of both CMs at varying flow rates of 5 ml/s, 2.5 ml/s and 1.0 ml/s (constant volume group: cVol). Therefore, bolus injection times of 1.0, 4.0 and 10.0 s were to be expected. The third injection protocol group employed injection of a constant small amount of CM (2 ml) at two different flow rates of 5.0 and 1.0 ml/s (micro volume group: mVol). Thus, the expected bolus injection times were 0.4 and 2.0 s, respectively. In all experiments the tube lines were initially flushed with saline according to the respective line volume. After the CM-injection a sufficient wash out of stained CM was reached by 25 ml saline in all protocols. The total amount of saline used in the various experiments was, therefore, the sum of the tube line volume and 25 ml of saline of the was out phase. As dimensions of tube-line systems differed considerably, especially between PS- and RP-type models, in an experimental setting the CovOpt and the UMiss injector were tested a second time. Here the PS-type CovOpt injector was equipped with the large volume tube-line system of the RP-type UMiss injector and vice versa.

All measurements were repeated on two different days resulting in a total of 151 measurements. Note that for each run overlapping injection protocols as well as the experimental measurements were performed only once. For a full summary of all employed protocols, please, refer to Table 4.

**Table 4:**
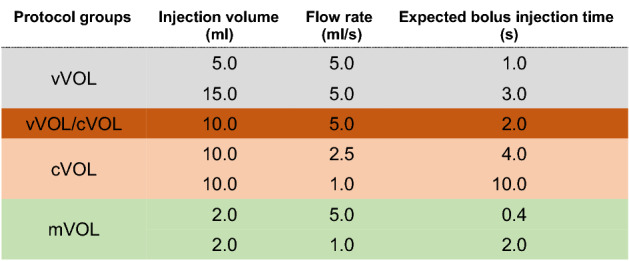
List of various prevalent injection protocols investigated in this study. Either the flow rate (vVol-protocol group: grey fields) or the injection volume (cVol-protocol group: red fields) were kept constant.

Note an overlap between vVOL and cVol- groups for injection of 10 ml CM at a flow rate of 5 ml/s (dark red fields). In addition, injection of very small CM-volumes was tested (mVol-protocol group: green fields).

### Computations and statistical assessment

We decided to use the boxcar function as an ideal reference for the shape of the expected photometric TC-curves. A boxcar function was fitted to the shape of each photometric TC-curve, where vertical straight lines were assumed in place of the discontinuity-points of the boxcar function (Fig. 4). For each TC-curve the Pearson correlation coefficient ($R_{P}$) was calculated to quantify the correlation with the fitted boxcar function (cBCF). The effective bolus injection time was derived from the interval length of the peak plateau phase of the fitted boxcar function and the difference to the expected bolus injection time (dBIT, unit: seconds) was calculated for each injection. Both parameters, i.e., cBCF and dBIT, were considered as rationally scaled. Testing was performed on all injection protocols (indicated by: [all]) and within protocol-groups (denoted by: [v,c,mVol]). Differences between the various injection protocols and groups concerning dBIT and cBCF were assessed using Kruskal–Wallis (KW-test) tests with Dunnett’s Modified Tukey–Kramer Pairwise Multiple Comparison Tests with conservative correction (Bonferroni) in the post hoc analysis in order to reduce bias from type 1 error (DTK-test). As all injectors varied considerably in technical aspects and tube line equipment, additionally, the influence of potential confounders on cBCF and dBIT was assessed using analysis of covariance (AnCova). A value of p < 0.05 was considered significant. Curve fitting and statistical computations were implemented using in-house developed scripts written in R^13^, thereby including packages: robustbase, DTK, car and vcd^14–17^.

## Supplementary information

Supplementary Information.

## References

[1] Tsuboyama T, Jost G, Pietsch H, Tomiyama N (2017). Comparison of power versus manual injection in bolus shape and image quality on contrast-enhanced magnetic resonance angiography. Investig. Radiol..

[2] Jost, G., Endrikat, J. & Pietsch, H. The impact of injector-based contrast agent administration on bolus shape and magnetic resonance angiography image quality. *Magn. Reson. Insights* **10**, 1178623X1770589 (2017). 10.1177/1178623x17705894 PMC5428122 28579796

[3] Kreitner, K.F.*, et al.* Systematische Analyse der Geometrie eines definierten kontrastmittelbolus—implikationen für die kontrastmittelverstärkte 3D-MR-angiographie thorakaler Gefäße. *RöFo: Fortschritte auf dem Gebiet der Röntgenstrahlen und der bildgebenden Verfahren* **177**, 646–654 (2005). 10.1055/s-2005-858091 15871079

[4] Indrajit I (2015). Pressure injectors for radiologists: a review and what is new. Indian J. Radiol. Imaging.

[5] Chaya A, Jost G, Endrikat J (2019). Piston-based vs peristaltic pump-based CT injector systems. Radiol. Technol..

[6] Seggern, D. Functions with a finite number of discontinuities. In *CRC Standard Curves and Surfaces.* 324 (CRC Press, Inc., Boca Raton, 1993).

[7] Laurent S, Elst LV, Muller RN (2006). Comparative study of the physicochemical properties of six clinical low molecular weight gadolinium contrast agents. Contrast Med. Mol. Imaging.

[8] Cruickshank Miller C (1924). The Stokes-Einstein law for diffusion in solution. Proc. R. Soc. Lond. A.

[9] Arduino Software. ARDUINO IDE (Arduino AG, 2020).

[10] WHATWG community. HTML living standard. Last updated 25 November 2019. Vol. 2019 (WHATWG community, 2019).

[11] Node.js. Node.js a JavaScript built runtime (2016).

[12] NetBeans IDE (supported by ORACLE). NetBeans IDE—overview (2016).

[13] R-Development CoreTeam. R: a language and environment for statistical computing (R Foundation for Statistical Computing, Vienna, Austria). ISBN 3–900051–07–0, 2015.

[14] Rousseeuw, P.*, et al.* Robustbase: basic robust statistics. R package version 0.92–5. https://CRAN.R-project.org/package=robustbase. Accessed 17 June 2014 (2015).

[15] Lau, M.K. DTK: Dunnett-Tukey-Kramer pairwise multiple comparison test adjusted for unequal variances and unequal sample sizes. R package version 3.5. https://CRAN.R-project.org/package=DTK. Accessed 22 September 2015 (2013).

[16] Meyer, D., Zeileis, A. & Hornik, K. VCD: visualizing categorical data (2017).

[17] Fox J, Weisberg S (2019). An {R} Companion to Applied Regression.

