# Supplementary material for: Disparity of time-contrast curves generated by various types of power injectors used in magnetic resonance imaging
Source: Sci Rep. 2020 Nov 11;10:19568. doi: 10.1038/s41598-020-76536-x (PMC7658966; doi:10.1038/s41598-020-76536-x)
Supplement: Supplementary file 1 — Supplementary Information. [file 41598_2020_76536_MOESM1_ESM.pdf]

Disparity of Time-Contrast Curves Generated by Various Types of Power Injectors Used in Magnetic Resonance Imaging

Doppler Marcus[1†], Moser Ewald[2], Klickovic Uros[1] and Nasel Christian[1,2†]

[1]Department of Radiology, University Hospital Tulln, Tulln, Austria

[2]Center for Medical Physics and Biomedical Engineering, Medical University of Vienna, Vienna, Austria

Corresponding Author:

Univ.Prof. Christian Nasel MSc MD PhD

University Hospital Tulln

Alter Ziegelweg 10

A – 3430 Tulln a.d. Donau

christian.nasel@meduniwien.ac.at

[†]These authors contributed equally to this work.

Supplementary Figure S1

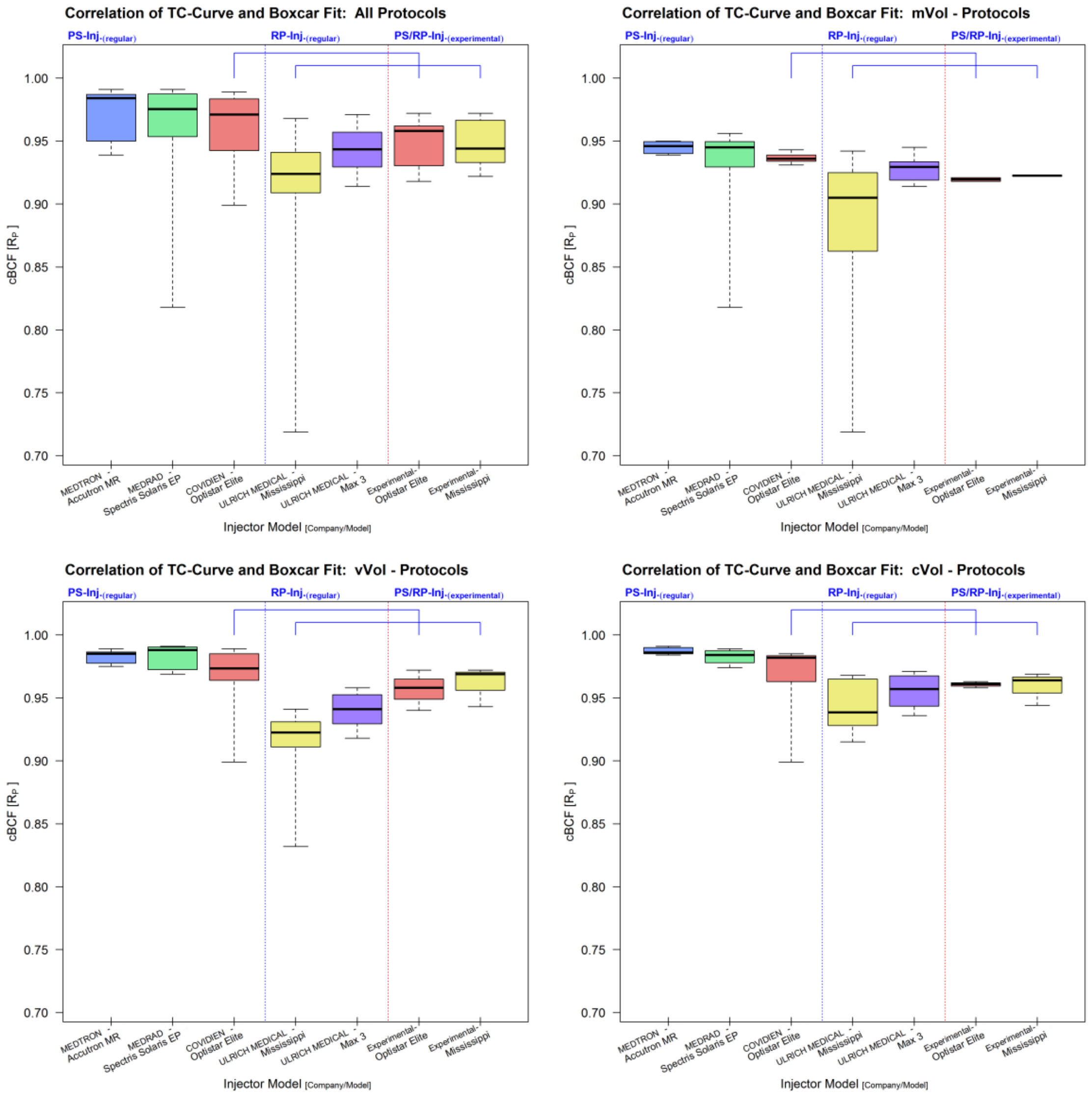

Supplementary Figure S1: Strength of cBCF was generally higher with PS-injectors in all tested protocols (1st row, left graph), though, this was less pronounced in injections of very small CM-volumes in the mVol-protocol group (1st row, right graph). This difference between PS- and RP-injectors reached statistical significance in vVol- (2nd row, left graph) and cVol- (2nd row, right graph) injection protocols, where common clinical MRI injection protocols were assessed. Experimental exchange of high and low volume line systems between a PS- and a RP-injector model, revealed some improvement with the RP-model, while the performance of the PS-model declined (horizontal brackets indicate injector ties). (For further interpretation of sub-graphs, please, also refer to figure 3.)

Supplementary Figure S2

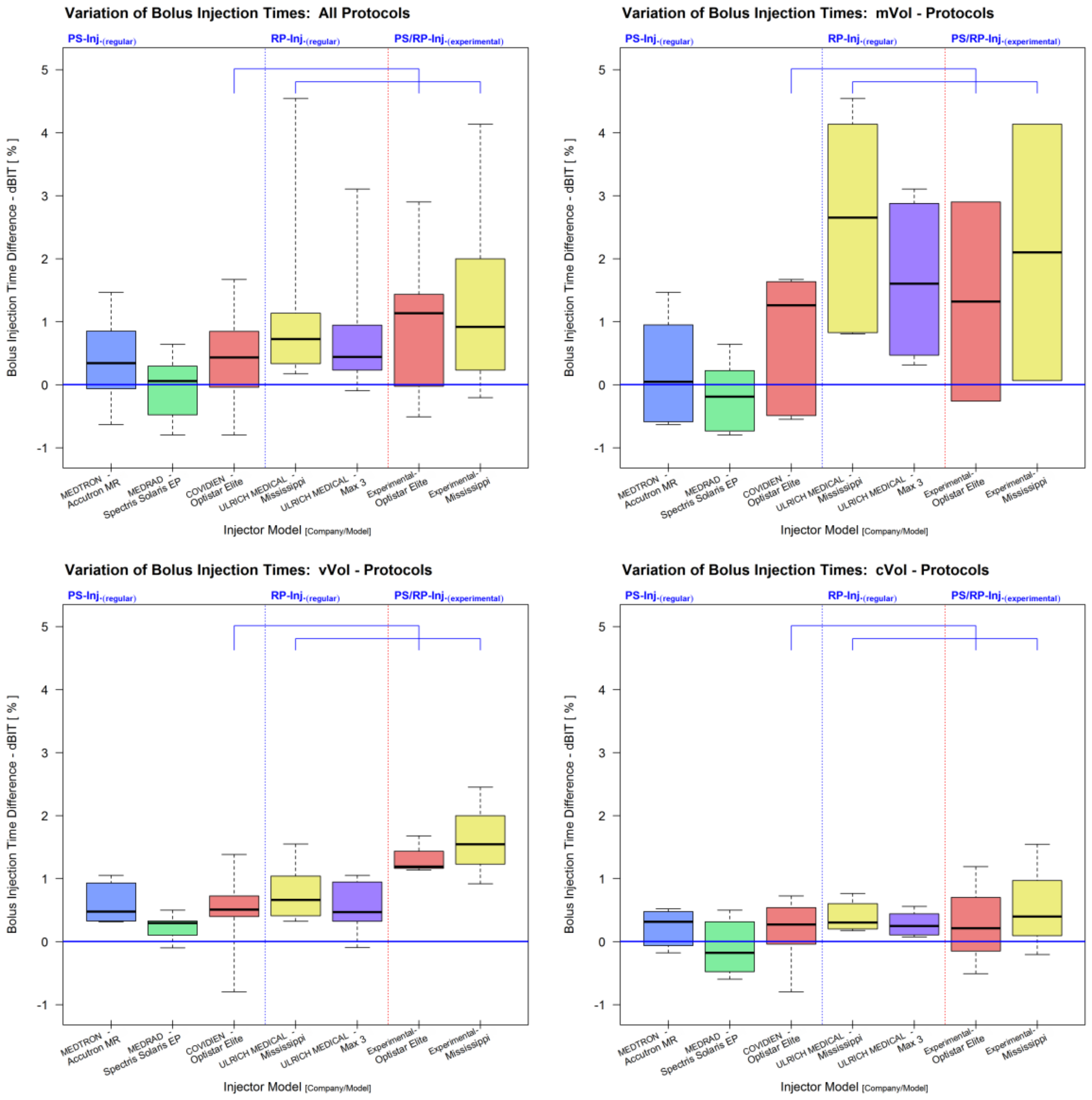

Supplemental Figure 2: True and expected injection times generally matched better in PS- than in RP-injectors (all-protocols; 1st row, left graph). Compared to injections of very small CM-volumes (mVol-protocol group; 1st row, right graph) variations of dBIT were clearly smaller with injections with either a constant flow rate of 5 ml/s (vVol-protocol group; 2nd row, left graph) or with common CM-volumes (cVol-protocol group; 2nd row, right graph). Experimental exchange of high and low volume tube lines between a PS- and a RP-injector model deteriorated dBIT in both injector types (horizontal brackets indicate injector ties). (Note that for reasons of comparability dBIT is given in percent of the expected injection time ! For further interpretation of sub-graphs, please, also refer to figure 4 in the main manuscript.)
